# Mechanisms and Clinical Translational Prospects of the Microbiota–Gut–Lung Axis in Connective Tissue Disease-Associated Interstitial Lung Disease

**DOI:** 10.3390/biom16101525

**Published:** 2026-10-20

**Authors:** Junxia Hu, Shiwen Ke, Lisha Mo, Guoshuang Zhu, Zenan Wu, Yingchong Chen

**Affiliations:** 1 School of Clinical Medicine, Jiangxi University of Chinese Medicine, Nanchang 330004, China; 2 School of Traditional Chinese Medicine, Jiangxi University of Chinese Medicine, Nanchang 330004, China; 3 Key Laboratory of Modern Chinese Medicinal Preparations, Ministry of Education, Jiangxi University of Chinese Medicine, Nanchang 330004, China

**Keywords:** connective tissue disease-associated interstitial lung disease, microbiota–gut–lung axis, gut microbiota, metabolic disturbance, immune imbalance, lung–gut synchronous therapy, integrated Chinese–Western prevention and treatment

## Abstract

Connective tissue disease-associated interstitial lung disease (CTD-ILD) is one of the most severe pulmonary complications of connective tissue diseases, featuring rapid progression and heterogeneous prognosis. Early recognition and prognostic prediction remain substantial clinical challenges. Accumulating evidence indicates that the microbiota–gut–lung axis participates in key pathological cascades, including autoimmune dysregulation, chronic inflammatory activation, and tissue fibrotic remodeling, which provides new insights into the pathogenesis of autoimmune-mediated lung diseases. This review systematically summarizes regulatory mechanisms of CTD-ILD involving gut dysbiosis, intestinal mucosal barrier impairment, disturbed metabolism of short-chain fatty acids, bile acids, and tryptophan, and systemic immune imbalance. We further compare evidence and pathological discrepancies of gut–lung–axis disturbance across CTD subtypes: systemic sclerosis-associated interstitial lung disease (SSc-ILD), rheumatoid arthritis-associated interstitial lung disease (RA-ILD), systemic lupus erythematosus-associated interstitial lung disease (SLE-ILD), idiopathic inflammatory myopathy interstitial lung disease (IIM-ILD), and primary Sjögren’s syndrome-associated interstitial lung disease (pSS-ILD). We also highlight the translational potential of gut–lung axis-related biomarkers for screening and assessment, summarize targeted interventions and integrated Chinese–Western therapeutic regimens, and pinpoint major evidence gaps and methodological limitations. This review refines the inter-organ regulatory framework of CTD-ILD and offers scientific references for mechanistic investigation, precise clinical evaluation, and novel therapeutic development.

## 1. Introduction

Connective tissue disorders (CTDs) represent a group of autoimmune diseases pathologically characterized by chronic non-infectious inflammation of systemic blood vessels and connective tissues, with multi-organ involvement throughout the body. The lungs are one of the most frequently affected target organs in CTDs [1]. Pathological lesions can invade the peribronchial connective tissues, pulmonary interstitium, alveolar walls, and perialveolar capillaries, leading to the development of interstitial lung disease (ILD), namely connective tissue disease-associated interstitial lung disease (CTD-ILD) [2].

CTD-ILD exhibits prominent clinical heterogeneity. ILD secondary to distinct CTD subtypes presents marked discrepancies in disease onset features, progression trajectories, radiological manifestations, and prognostic outcomes, with substantial inter-individual variability in clinical profiles [3]. Nevertheless, multiple challenges persist in the clinical management of CTD-ILD. Non-invasive biomarkers for early-stage screening, accurate disease assessment, prediction of disease progression, and warning of acute exacerbations remain lacking. Consequently, early diagnosis, dynamic monitoring of disease progression, and precise risk stratification for patients are difficult to achieve in clinical practice. Most patients have already developed irreversible pulmonary interstitial damage at the time of definitive diagnosis. Current therapeutic regimens are mainly based on glucocorticoids, immunosuppressants, and anti-fibrotic agents. However, these strategies suffer from insufficient therapeutic specificity and prominent adverse effects. They can only moderately slow the decline in pulmonary function, cannot fundamentally halt fibrotic progression, and fail to substantially improve the long-term prognosis of patients [4]. Accordingly, in-depth exploration of novel pathogenic mechanisms underlying CTD-ILD and the identification of safe and efficient targeted therapeutic targets represent high-priority research imperatives that urgently need to be addressed in this field.

The microbiota–gut–lung axis (referred to as the gut–lung axis) constitutes a critical bidirectional regulatory system for distant organs in a host. Relying on gut microbial communities, intestinal mucosal barriers, the circulatory system, and systemic immune system, it mediates dynamic signal crosstalk and functional modulation between the intestine and the lung, serving as a core physiological system for sustaining immune homeostasis and inflammatory balance in both the respiratory tract and intestinal tract [5]. Under physiological conditions, the structure of gut microbiota remains relatively stable. By modulating host immune responses, secreting functional metabolites, restraining pathological bacterial translocation, and tuning systemic inflammatory status, gut microbiota contribute to the homeostasis of the pulmonary microenvironment and enhance the capacity of lung tissues to resist inflammatory stimuli and pathological injuries [6]. Under pathological conditions, gut dysbiosis, intestinal leakage induced by impaired intestinal mucosal barrier, and perturbed microbial metabolic profiles can break host immune tolerance, drive systemic low-grade chronic inflammation and autoimmune aberrancies, and further mediate pulmonary inflammatory activation and fibrotic remodeling. Cumulative evidence has demonstrated that gut–lung axis dyshomeostasis is extensively involved in the pathological progression of multiple chronic inflammatory and autoimmune diseases, and is highly correlated with autoimmune dysregulation and organ fibrotic lesions.

## 2. Core Mechanisms of the Microbiota–Gut–Lung Axis Mediating the Pathogenesis and Progression of CTD-ILD

### 2.1. Gut Dysbiosis and Systemic Immune Imbalance

**Figure 1:**
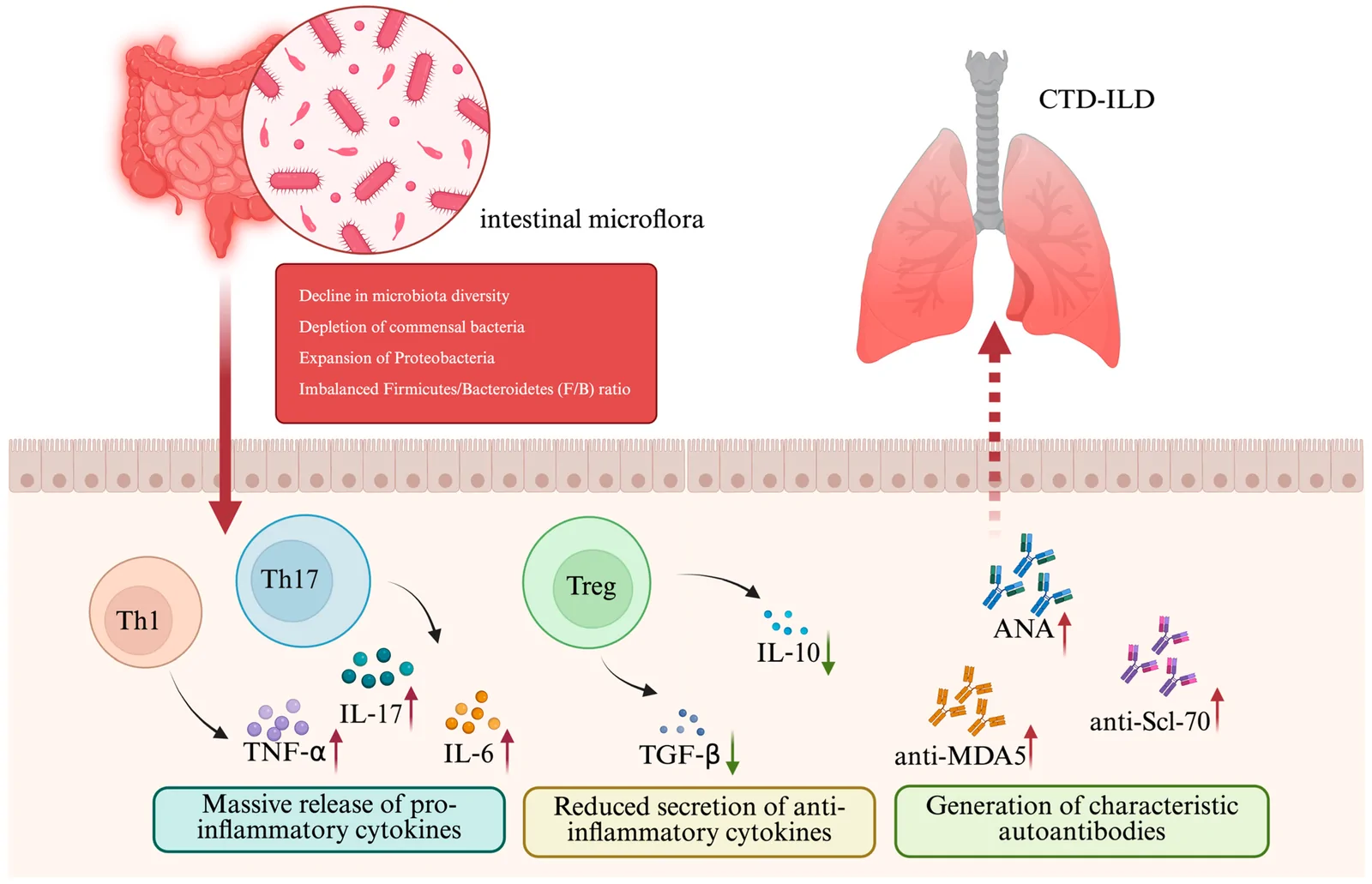
Schematic overview of gut–lung axis-mediated immunopathogenesis in CTD-ILD. Intestinal dysbiosis, characterized by reduced microbial diversity, commensal depletion, Proteobacteria expansion, and imbalanced Firmicutes/Bacteroidetes ratio, disrupts host immune tolerance and drives systemic autoimmunity, eventually leading to secondary pulmonary interstitial injury. This process is hypothesized to represent an important early trigger for CTD-ILD onset. Red upward arrows denote increased abundance or expression; green downward arrows indicate reduced secretion or functional impairment.

As the largest microecosystem of the human body, the gut microbiota serve as a central hub for maintaining host immune homeostasis and modulating systemic inflammatory responses. Their structural perturbation and homeostatic disruption represent key initiating factors driving the pathogenesis and progression of autoimmune diseases [7]. Gut microbiota can modulate intestinal and pulmonary immune systems via local and long-term crosstalk [8]. Under physiological conditions, gut microbiota maintain intestinal mucosal and systemic immune homeostasis through multiple pathways, including regulating the balance between Treg and Th17 cells, inducing the differentiation of tolerogenic dendritic cells, and ensuring normal secretion of secretory immunoglobulin A (sIgA) [9]. Upon the onset of gut dysbiosis, microecological remodeling occurs, manifested by reduced microbial diversity, depletion of commensal bacteria, expansion of Proteobacteria, and an imbalanced Firmicutes/Bacteroidetes ratio. These alterations break host immune tolerance via multiple immune-disordered mechanisms, drive systemic autoimmunity, and consequently facilitate the development of secondary pulmonary interstitial injury [10] (Figure 1).

The immune dysregulation mediated by such microecological imbalance is manifested as follows: gut dysbiosis bidirectionally disturbs host immune homeostasis. On the one hand, it suppresses Treg-cell differentiation, leading to insufficient secretion of anti-inflammatory cytokines, including IL-10 and TGF-β, and attenuates host anti-inflammatory protective capacity. On the other hand, it drives aberrant polarization of Th1 and Th17 cells, triggers the robust release of pro-inflammatory mediators such as IL-6, IL-17, and TNF-α, and induces systemic inflammatory disturbance [11,12]. Meanwhile, dysregulated gut microbiota can facilitate the generation of CTD-specific autoantibodies, including ANA, anti-Scl-70, and anti-MDA5, through aberrant autoantigen presentation, molecular mimicry, and other mechanisms [13]. These autoantibodies can target and deposit in pulmonary interstitial tissues via blood circulation, trigger local antigen-specific immune injury in the lung, and drive the progression of interstitial lung lesions. Accumulating studies indicate that patients with CTD frequently exhibit marked gut dysbiosis, mainly manifested as reduced microbial diversity, decreased abundance of beneficial commensal bacteria, and excessive enrichment of pro-inflammatory pathogenic bacteria. Such alterations further break systemic immune tolerance and provide a sustained immunological basis for the initiation and progression of pulmonary lesions [14,15]. Nevertheless, there is no consensus regarding the key gut microbiota that mediate tissue injury across distinct CTD-ILD subtypes. Evidence supporting the correlations between specific gut microbes, autoantibodies, and pulmonary interstitial injury remains to be further validated. Taken together, it is reasonable to speculate that systemic immune dysregulation triggered by gut dysbiosis serves as an important early trigger for the initiation of CTD-ILD.

### 2.2. Intestinal Barrier Impairment and Pulmonary Inflammatory Cascade Reactions

**Figure 2:**
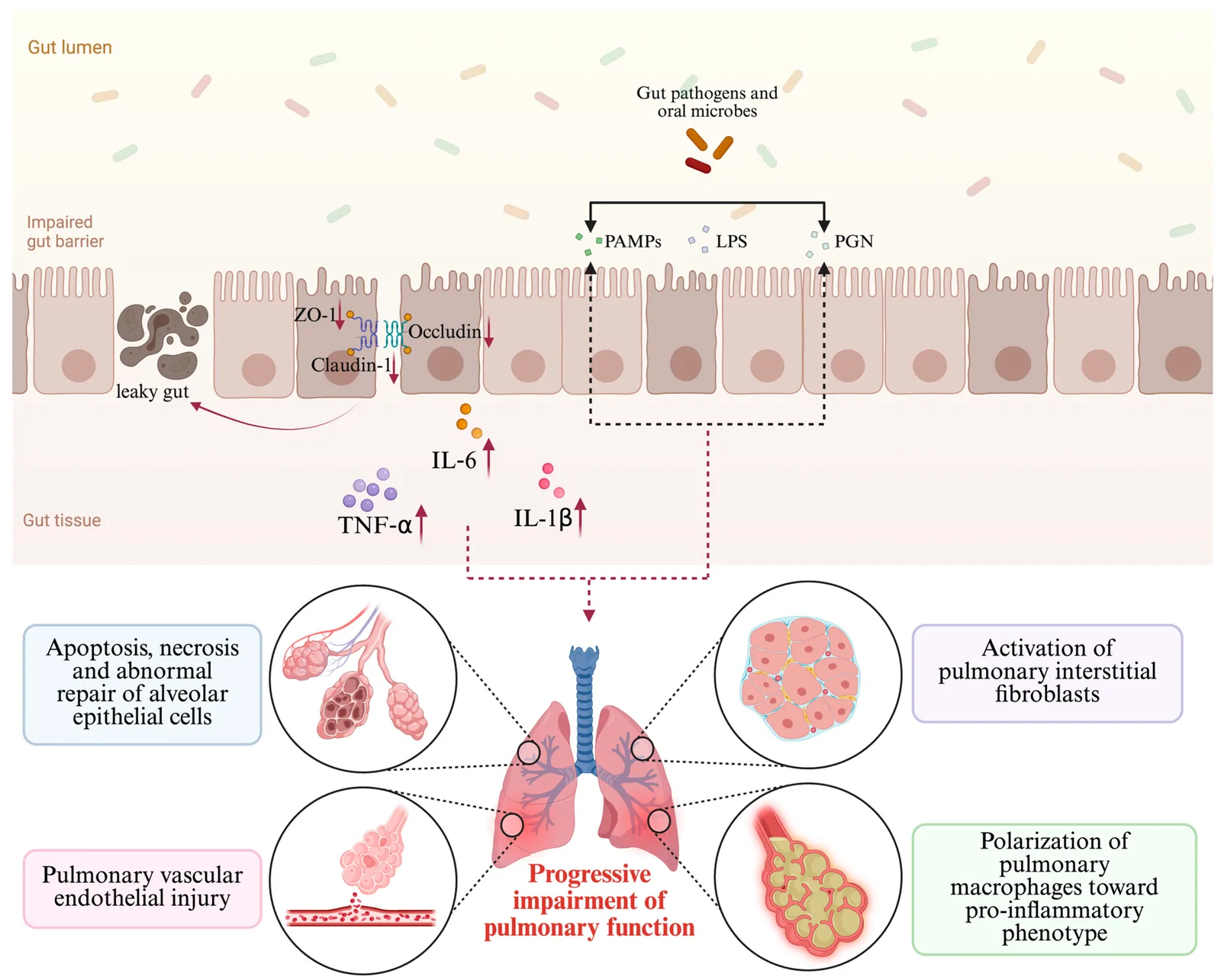
Schematic overview of pulmonary inflammatory cascades secondary to intestinal barrier impairment in CTD-ILD. Gut dysbiosis reduces tight-junction protein expression to induce “leaky gut”. Translocated luminal PAMPs and gut-derived pro-inflammatory mediators enter systemic circulation and target lung tissue. These pathogenic triggers elicit multiple pulmonary pathological alterations, including epithelial and endothelial injury, macrophage pro-inflammatory polarization, and fibroblast activation, leading to interstitial tissue remodeling and progressive decline in pulmonary function.

The intestinal mucosal barrier serves as the core physical and functional barrier that segregates gut microecology from the host circulatory system, and acts as a critical intermediate mediator for crosstalk regulation within the gut–lung axis [16]. Under physiological conditions, the structurally intact intestinal mucosal barrier can effectively block gut microbiota and toxic metabolites from entering the bloodstream, maintaining host immune balance and inflammatory homeostasis. Nevertheless, gut dysbiosis can directly disrupt the structural integrity and physiological function of the intestinal barrier, leading to reduced expression of intestinal epithelial tight-junction proteins (ZO-1, Occludin, Claudin-1) and abnormally elevated epithelial permeability, which triggers the typical pathological feature of “intestinal leakage” [17,18]. Intestinal barrier injury acts as a critical pathological bridge for the transmission of aberrant intestinal signals to the lung and the induction of pulmonary interstitial inflammatory lesions. When the intestinal barrier is impaired, luminal PAMPs, incompletely degraded inflammatory mediators, and toxic microbial metabolites can penetrate the intestinal epithelial barrier and continuously leak into the peripheral circulation. These leaked substances mainly include gut microbial structural components such as lipopolysaccharide and peptidoglycan, accompanied by multiple pro-inflammatory cytokines including TNF-α, IL-6, and IL-1β released by local intestinal inflammatory cells [19]. These gut-derived pathogenic factors undergo systemic ectopic dissemination via blood circulation and are continuously delivered to lung tissues. Long-term exposure of the lung to gut-derived inflammatory stimuli disrupts pulmonary innate immune homeostasis and lays a critical pathological foundation for sustained inflammatory injury and tissue remodeling in the pulmonary interstitium (Figure 2).

Circulating gut-derived pathogenic factors can mediate pulmonary injury through multiple targets and pathways, amplify the inflammatory cascade step-by-step, and drive persistent progression of ILD. On the one hand, circulating toxic mediators can directly damage alveolar epithelial cells, induce alveolar epithelial apoptosis, necrosis, and aberrant repair, and disrupt normal alveolar architecture. Meanwhile, they activate alveolar capillary endothelial cells, aggravate pulmonary vascular endothelial injury and barrier leakage, and exacerbate pulmonary inflammatory exudation and tissue edema [20,21,22]. On the other hand, ectopically deposited inflammatory signals can induce pro-inflammatory polarization of pulmonary macrophages, which continuously secrete abundant pro-inflammatory and pro-fibrotic cytokines to further amplify local inflammatory responses. In addition, the persistent pulmonary inflammatory microenvironment can activate pulmonary interstitial fibroblasts, trigger their aberrant proliferation and transformation into myofibroblasts, and promote massive extracellular matrix deposition. These events ultimately result in interstitial structural remodeling and fibrotic hyperplasia, progressively deteriorate pulmonary function, and drive CTD-ILD to progress from inflammatory injury toward irreversible fibrotic lesions [23].

Beyond the cellular cascade described above, innate immune sensing and neutrophil-and endothelium-centered mechanisms further amplify gut-originated systemic inflammation and bridge it to pulmonary fibrosis. Following intestinal barrier impairment, translocated microbial nucleic acids engage endosomal TLR7/8 in plasmacytoid dendritic cells, monocytes/macrophages, and neutrophils, driving MyD88–IRF7-dependent type I interferon (IFN-α/β) production and an interferon-stimulated-gene cascade that sustains systemic low-grade inflammation and autoimmune activation [24]. Concurrently, primed neutrophils form extracellular traps (NETs) composed of DNA, histones, myeloperoxidase (MPO), and neutrophil elastase, which damage alveolar epithelial and endothelial cells and, via TGF-β activation, promote myofibroblast differentiation. These LPS-, IFN-, and NET-associated mediators also activate pulmonary endothelial cells—increasing vascular permeability, adhesion molecule expression (ICAM-1, VCAM-1, and E-selectin), and impairing nitric oxide signaling—thereby facilitating inflammatory cell recruitment and fibroblast activation [25]. Collectively, these amplifying mechanisms form an innate immune–neutrophil–endothelial axis that strengthens the gut–lung vicious cycle and drives progressive fibrotic remodeling in CTD-ILD.

### 2.3. Distal Targeted Regulation Mediated by Gut Microbial Metabolites

**Figure 3:**
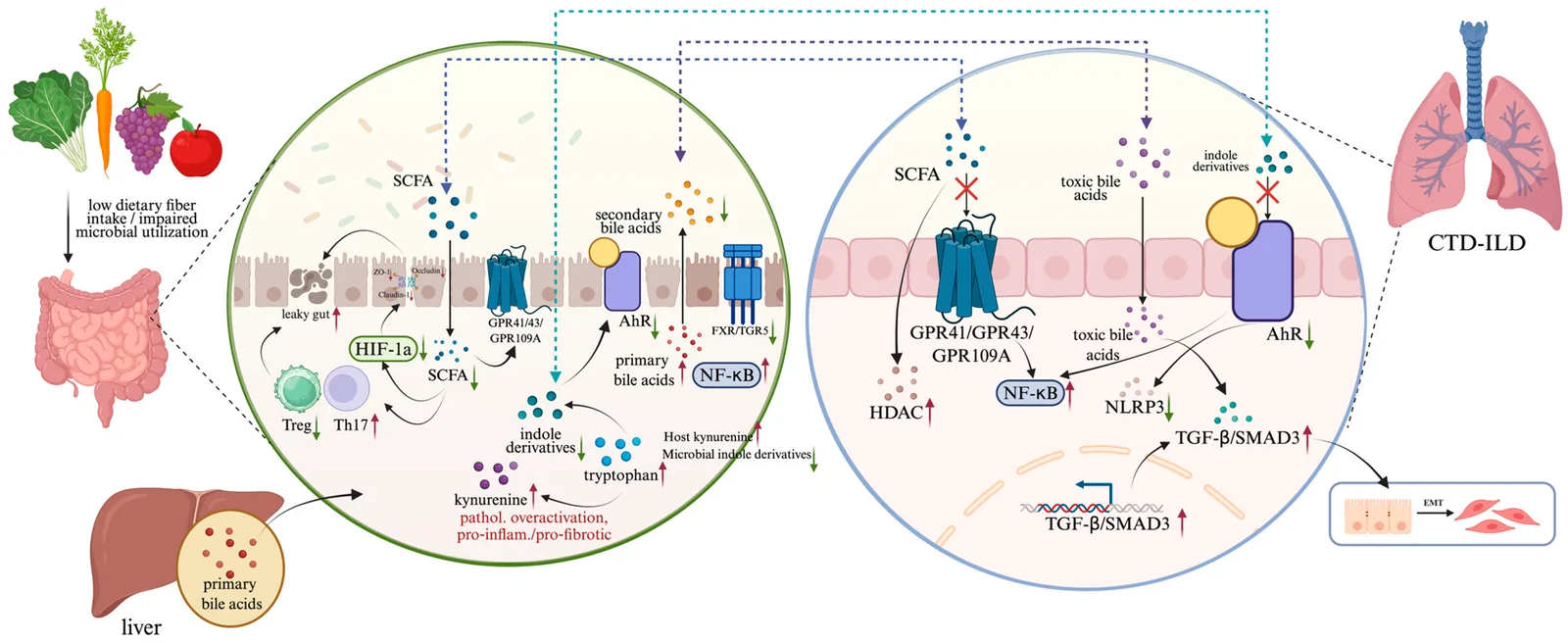
Schematic diagram of cross-organ regulation mediated by gut microbial metabolites in the gut–lung axis. Intestinal microbiota biotransform dietary substrates into SCFAs, secondary bile acids, and tryptophan-derived indole metabolites. Under physiological conditions, metabolites cross intact intestinal epithelium for distant organ regulation; under dysbiosis-related barrier damage, increased intestinal permeability enhances metabolite translocation. These circulating metabolites target GPR41/43/GPR109A, TGR5/FXR, and AhR receptors in the lung, modulate NF-κB and TGF-β/Smad signaling, and balance Treg/Th17 immune responses, thereby regulating pulmonary immune homeostasis and participating in CTD-ILD pathological progression.

The metabolic activity of gut microbiota is not restricted to the intestine itself. A variety of small-molecule substances synthesized by gut microbiota can penetrate the intestinal barrier, participate in systemic signal transduction through blood and lymphatic circulation, target receptors or signaling axes of distal organs, and achieve cross-organ homeostasis regulation or disease intervention. They serve as core functional mediators of the gut–lung axis (Figure 3). Microbial metabolites can cross anatomical barriers to reshape distal microenvironments. Compared with the local effects of gut microbiota, they exhibit advantages including broad action range, high stability, and favorable targeting properties. Among them, short-chain fatty acids, bile acids, and tryptophan metabolites are pivotal mediators regulating the pulmonary microenvironment and mediating gut-lung crosstalk, and are widely involved in maintaining pulmonary physiological homeostasis as well as the repair and modulation of pathological injury [26].

#### 2.3.1. Short-Chain Fatty Acids (SCFAs)

SCFAs mainly include acetate, propionate, and butyrate, which are small-molecule signaling metabolites produced by anaerobic gut bacteria via fermentation of insoluble dietary fibers [27]. As core endogenous humoral mediators governing gut–lung axis crosstalk, SCFAs can enter the systemic circulation through the portal vein and exert long-distance targeting effects on lung tissues, executing multiple distal regulatory functions including intestinal barrier protection, immune homeostasis modulation, and anti-inflammatory as well as anti-fibrotic activities [28]. Accumulating studies have demonstrated that SCFAs exert broad-spectrum protective effects against pulmonary inflammation and fibrotic lesions through diverse pathways [29,30,31]. In terms of distal targeting regulatory mechanisms, SCFAs can upregulate the expression of intestinal epithelial tight-junction proteins and reduce epithelial permeability, thereby preserving the structural and functional integrity of the intestinal mucosal barrier, inhibiting intestinal leakage, and blocking the entry of gut microbial components and toxic metabolites into the bloodstream. These effects fundamentally prevent ectopic transmission of gut-derived inflammatory signals to the lung. Meanwhile, SCFAs maintain systemic immune homeostasis by balancing the Treg/Th17 axis, promote the proliferation and activation of regulatory T-cells, suppress pro-inflammatory responses induced by excessive Th17-cell activation, and ameliorate systemic immune dysregulation [5]. Furthermore, SCFAs modulate inflammatory and immune responses through dual pathways: activating G-protein-coupled receptors (GPCRs) and inhibiting histone deacetylases (HDACs). They regulate the transcriptional expression of inflammation-related genes, restrain amplification of the inflammatory cascade, and alleviate systemic inflammatory injury [28,29].

Existing research has confirmed that SCFAs can block gut-derived inflammatory signals from reaching the lung at the source via distal targeting pathways of the gut–lung axis. They simultaneously modulate pulmonary immune dysregulation and suppress inflammatory responses as well as interstitial fibrosis, exhibiting the advantages of safety, high efficiency, and favorable targeting properties [32]. As a specific interstitial lung disease secondary to connective tissue diseases, CTD-ILD shares highly homologous pathological features of lung injury with idiopathic ILD. Therefore, it is reasonable to deduce that gut–lung axis imbalance mediated by gut microbiota and SCFAs may synergistically accelerate CTD-ILD progression by superimposing systemic autoimmune dysregulation. This mechanism remains to be further verified by subsequent dedicated studies.

#### 2.3.2. Bile Acids

Bile acids are steroidal carboxylic acid-derived active molecules generated from cholesterol metabolism. Conventionally, their major physiological function is to facilitate intestinal lipid digestion and absorption [33]. Through in-depth research on gut–lung axis mechanisms, bile acids are recognized as critical humoral mediators that rely on gut microbiota-driven metabolic remodeling to mediate cross-organ signal communication, and participate in the pathological processes of pulmonary inflammation and fibrosis via distal regulation. Gut microbiota modify primary bile acids to produce secondary bile acids, which are extensively involved in the differentiation of innate and adaptive immune cells as well as the balance of cytokine secretion, constituting one of the core metabolic regulatory pathways within the gut–lung axis [34]. Bile acids exert biological effects mainly through the membrane receptor TGR5 and the nuclear receptor FXR [35]: activation of the FXR pathway suppresses pro-inflammatory pathway activation in the alveoli, pulmonary interstitium, and intestine, and reduces the release of pro-fibrotic factors. TGR5 signaling modulates macrophage polarization and inhibits amplification of inflammatory cascades. Jointly, they regulate chronic pulmonary inflammation, interstitial collagen deposition, and fibrotic progression, providing potential regulatory mechanisms for pathological injury in CTD-ILD [36].

Notably, patients with systemic sclerosis (SSc) frequently suffer from gastroesophageal reflux and microaspiration [37,38]. This feature endows bile acids with dual injury-mediating mechanisms in the pathogenesis of SSc-ILD. On the one hand, as gut-derived metabolic signals, bile acids remotely modulate the pulmonary microenvironment via the gut–lung circulation. On the other hand, bile acids refluxed into the airways and alveoli can directly trigger microaspiration-associated lung injury, locally aggravate alveolar epithelial damage and interstitial chronic inflammation [39], and may serve as a unique pathological factor for SSc-ILD. Nevertheless, CTD-ILD exhibits strong inter-subtype heterogeneity and substantial phenotypic differences. Current studies regarding bile acid profiles, receptor pathways, and upstream–downstream regulatory networks in patients with CTD-ILD remain limited. At present, bile acid dysregulation can only be regarded as a potential target for interpreting the pathogenesis of CTD-ILD, especially SSc-ILD. Further high-quality basic and clinical studies are required to verify its exact regulatory effects and clinical implications.

#### 2.3.3. Tryptophan

Cumulative evidence demonstrates that tryptophan, an essential amino acid and key gut-derived signaling molecule, mediates long-distance crosstalk between the intestinal microenvironment and pulmonary immune homeostasis [32]. Endogenous tryptophan is metabolized mainly via three pathways: the gut microbiota-driven indole pathway, the host kynurenine pathway, and the host serotonin biosynthetic pathway [40,41]. Among them, the kynurenine pathway accounts for 90–95% of systemic tryptophan metabolic flux. It is primarily initiated by constitutive TDO2 and inflammation-inducible IDO1, and is extensively involved in autoimmune regulation, chronic inflammatory responses, and tissue fibrotic remodeling. Only 1–3% of tryptophan enters the serotonin synthesis pathway, where it is catalyzed by TPH1/2 to produce serotonin, which exerts diverse cellular biological functions by acting on G-protein-coupled receptors. In contrast, luminal free tryptophan unabsorbed by the small intestine can be metabolized by commensal gut bacteria to generate bioactive products including indole and indole-3-propionic acid (IPA). These metabolites protect the intestinal barrier and suppress excessive inflammatory responses via activation of the AhR signaling pathway [42,43,44]. By competing for common substrates and cross-regulating downstream signals, the three pathways synergistically maintain immune and epithelial barrier homeostasis within the gut–organ axis. Once this metabolic balance is disrupted, dysregulated tryptophan metabolism serves as an upstream initiating factor that drives the gut–lung axis to shift from physiological homeostasis toward pathological injury, thereby triggering gut-originated pulmonary injury and fibrosis.

Inflammatory stimuli can markedly induce aberrant overexpression of IDO1, accelerate the conversion of tryptophan to kynurenine, and result in a significantly elevated Kyn/Trp ratio. Existing studies indicate that an increased Kyn/Trp ratio is closely associated with elevated disease activity, declined pulmonary function, and poor prognosis in multiple autoimmune diseases, and can serve as a critical metabolic biomarker reflecting systemic immune dysbalance and pathological injury [45,46]. Significant reduction in serum L-tryptophan levels has been observed in animal models of systemic sclerosis and fibrotic interstitial lung disease [47]. Specific studies clarifying how tryptophan metabolic disorders mediate pulmonary interstitial injury through the gut–lung axis across distinct CTD-ILD subtypes remain scarce, representing a critical direction to be further explored in this field.

### 2.4. Vicious Cycle of Gut–Lung Axis Crosstalk: A Hypothetical Regulatory Framework

**Figure 4:**
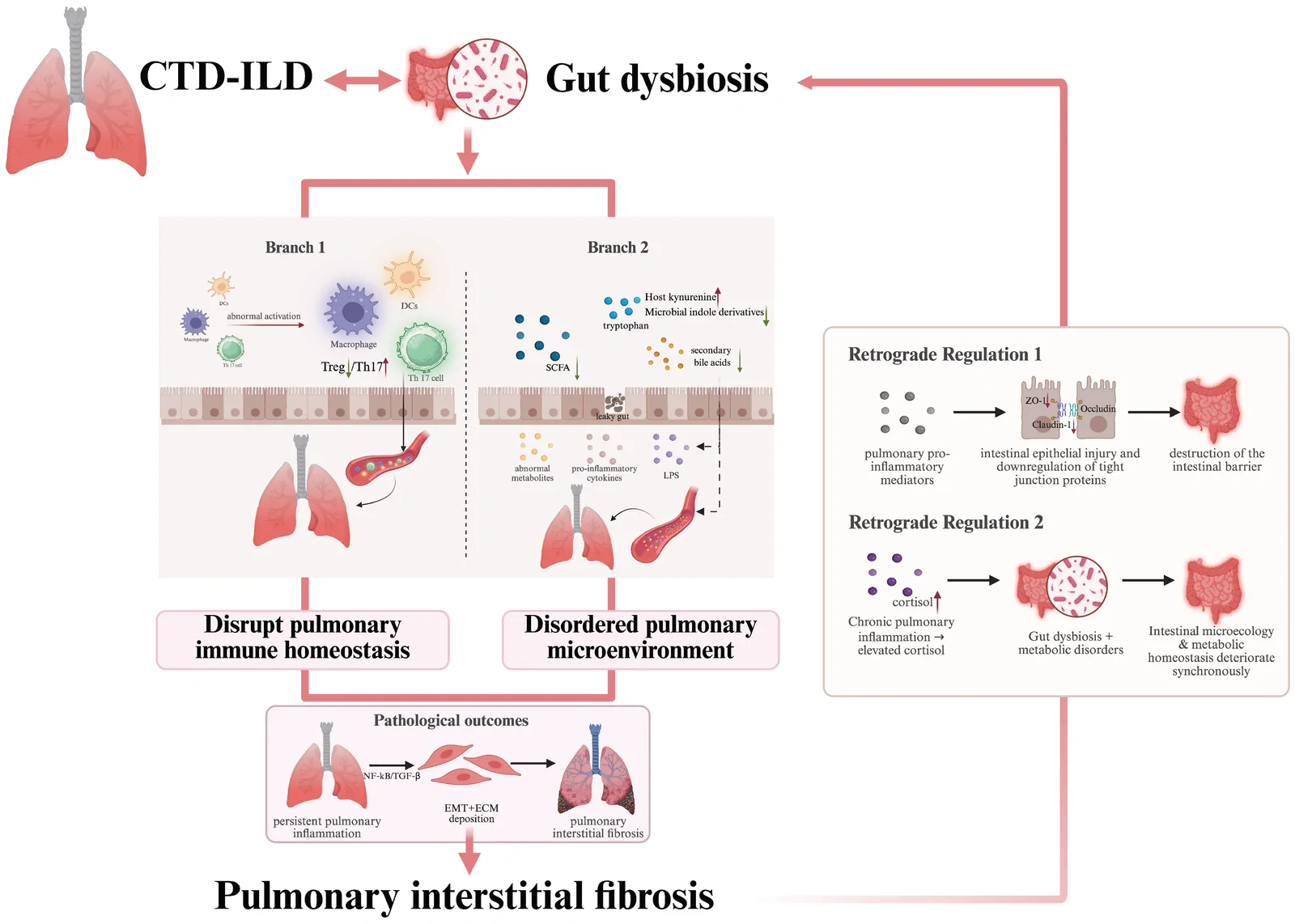
Schematic illustration of the bidirectional vicious cycle hypothesis of the gut–lung axis in CTD-ILD. CTD-related pathological stimuli initiate gut dysbiosis, which drives pulmonary injury through two core pathways. Branch 1 represents immune dysregulation: activated dendritic cells and macrophages induce excessive Th17 expansion and pulmonary migration, disrupting lung immune homeostasis. Branch 2 represents intestinal barrier and metabolic dysfunction: reduced SCFAs and disordered tryptophan as well as bile acid metabolism cause intestinal leakage, allowing translocation of LPS and inflammatory metabolites to trigger chronic pulmonary inflammation, fibroblast activation, EMT, ECM deposition, and interstitial fibrosis. In turn, pulmonary inflammation retrogradely impairs intestinal barrier integrity and aggravates gut microbial disturbance via inflammatory mediators and elevated cortisol. The bidirectional gut–lung feedback loop continuously amplifies pathological damage and sustains CTD-ILD progression.

Based on previous evidence regarding gut microecological imbalance, intestinal barrier dysfunction, systemic immune dysregulation, and pulmonary fibrotic pathology, this section puts forward a working hypothesis of a gut–lung bidirectional vicious cycle to systematically illustrate the potential closed-loop regulatory rules for CTD-ILD progression mediated by the gut–lung axis. The intestinal and pulmonary mucosal immune systems share high structural and functional homology, as well as core immune cell subsets and inflammatory regulatory networks, which lay anatomical and physiological foundations for their distal crosstalk and bidirectional modulation. By synthesizing existing research findings, a cascade-driven closed regulatory loop underlying gut–lung axis-mediated CTD-ILD progression can be summarized (Figure 4).

During disease progression, gut dysbiosis acts as the key upstream initiator that triggers pathological gut–lung crosstalk. Disturbed gut microecology can directly destroy the structural integrity of the intestinal mucosa and lead to abnormally elevated intestinal barrier permeability. Upon impairment of intestinal barrier integrity, luminal LPS, PAMPs, and aberrant microbial metabolites penetrate into the peripheral circulation in large quantities, break host immune homeostasis, and trigger excessive activation of systemic immune-inflammatory responses. After being disseminated to lung tissues via blood circulation, these systemic inflammatory signals induce multi-dimensional pulmonary pathological injury. On the one hand, they cause damage and apoptosis of alveolar epithelial cells. On the other hand, they drive pro-fibrotic phenotypic activation of pulmonary macrophages and aberrant proliferation of lung fibroblasts, ultimately resulting in sustained pulmonary inflammatory responses and excessive extracellular matrix deposition, and driving persistent progression of pulmonary fibrosis. More importantly, this regulatory pathway is not a unidirectional inflammatory cascade, but exhibits a self-amplifying vicious cycle effect: progressive pulmonary inflammation and fibrotic lesions can retroact on the intestine through systemic inflammatory circulation and neuro-immune-endocrine regulatory pathways, further disturbing gut microecology, aggravating intestinal barrier injury, and disrupting intestinal metabolic homeostasis. Consequently, a bidirectional pathological closed loop is formed: gut dysbiosis–intestinal barrier disruption–systemic immune dysregulation–pulmonary inflammation and fibrosis progressive deterioration of intestinal injury. This hypothetical framework summarizes the core pathological logic of bidirectional gut–lung axis crosstalk, explains the chronic and progressive clinical features of CTD-ILD, and clarifies the intrinsic causal relationship between gut microecological disturbance and pulmonary fibrosis progression. Meanwhile, it provides a reliable theoretical model and research insights for future basic mechanistic studies targeting the gut–lung axis in CTD-ILD and the exploration of novel clinical intervention strategies.

## 3. Commonalities and Differences of the Gut–Lung Axis Among Distinct CTD Subtypes

CTD-ILD represents the most prevalent severe pulmonary complication of CTD. As the core pathway mediating interactions among the intestinal mucosal barrier, gut microbiota, intestinal immunity, and pulmonary inflammation/fibrosis, the gut–lung axis plays a pivotal role in the pathogenesis of various CTD-ILD subtypes (Table 1). Nevertheless, remarkable subtype-specific characteristics exist due to distinct autoimmune signatures and target organ injury profiles across different subtypes.

### 3.1. SSc-ILD

SSc is a rare connective tissue disease characterized by autoimmune dysregulation, microvascular injury, and multi-organ fibrosis. It can involve multiple organs including skin, gastrointestinal tract, lung, heart, and kidney, among which gastrointestinal manifestations are highly heterogeneous [48]. SSc-ILD represents the leading cause of death among SSc patients. Pulmonary interstitial lesions occur in 40–60% of SSc patients, and approximately one-third of patients develop progressive and irreversible decline in pulmonary function, which eventually progresses to respiratory failure [49]. Among various CTD subtypes, SSc serves as an optimal disease model for illustrating gut–lung axis crosstalk, mainly due to its unique pathological feature of pan-digestive-tract fibrosis. More than 70% of SSc patients exhibit characteristic esophageal smooth-muscle fibrosis, esophageal dysmotility, reduced gastrointestinal motility, and small intestinal bacterial overgrowth, which further trigger gastroesophageal reflux and occult microaspiration [50]. These events continuously induce chronic micro-injury in distal airways and pulmonary interstitium and disrupt pulmonary microecological homeostasis [51], providing critical pre-pathological conditions for the initiation and persistent progression of pulmonary interstitial fibrosis. Structural remodeling and motility disorders in the digestive tract of SSc patients can further disrupt intestinal tight-junction architecture, resulting in increased intestinal barrier permeability, gut dysbiosis, and disturbed microbial metabolism [52]. Following intestinal barrier disruption, abundant endotoxins, peptidoglycans, and aberrant microbial metabolites are released into the bloodstream [53]. Via circulatory pathways of the gut–lung axis, these factors remotely regulate pulmonary inflammation and tissue repair, persistently activate lung fibroblasts, promote aberrant collagen deposition in the lung, and mediate irreversible progression of pulmonary interstitial fibrosis [53].

Immune imbalance constitutes the core pathogenesis of SSc, featured by persistent over-activation of Th17 cells and defective immunosuppressive function of Treg cells [54]. Aberrantly activated gut-derived Th17 cells can migrate toward the pulmonary interstitium, continuously amplify chronic pulmonary inflammation, and aggravate alveolar structural destruction as well as interstitial remodeling [55]. As an initiating and hallmark pathological alteration of SSc, microvascular injury affects microcirculation in both the intestine and lung and leads to local tissue ischemia and hypoxia [56]. On the one hand, it aggravates intestinal mucosal injury, barrier leakage, and gut dysbiosis, and continuously amplifies the output of gut-derived inflammatory signals. On the other hand, it induces aberrant repair in lung tissues and promotes secondary fibrotic progression. Consequently, a distinctive pathological vicious cycle of “microvascular injury–intestinal barrier dysfunction–pulmonary interstitial fibrosis” is established and continuously intensified in SSc-ILD [57,58,59]. Overall, systemic acute inflammatory responses remain relatively moderate in SSc. Disease progression is not dominated by robust inflammatory bursts, but relies on sustained activation of fibrosis-related signaling hubs to drive progressive and irreversible multi-organ fibrotic deterioration. In summary, the unique pan-digestive-tract fibrosis, reflux-associated microaspiration, and pulmonary microecological disturbance in SSc lay the structural foundation for aberrant gut–lung axis activation. The synergistic interplay among gut dysbiosis, Th17/Treg immune imbalance, and systemic microangiopathy forms a stable pathogenic loop of the gut–lung axis that dominates the onset and progression of SSc-ILD. Key regulatory targets and microbial biomarkers within this axis hold promise as important research directions for further mechanistic exploration, early warning, and novel targeted interventions for SSc-ILD in future studies.

### 3.2. RA-ILD

RA is a chronic autoimmune connective tissue disease that predominantly manifests as symmetric erosive synovitis. Patients aberrantly produce characteristic autoantibodies, including rheumatoid factor and anti-cyclic citrullinated peptide (anti-CCP) antibodies, which mediate persistent low-grade systemic chronic inflammation and result in progressive joint destruction [60]. Beyond typical joint manifestations, RA can affect multiple systemic organs. RA-ILD is its most representative extra-articular complication with poor prognosis. Characterized by insidious onset, recurrent chronic interstitial pulmonary inflammation, and progressive fibrosis, RA-ILD constitutes a critical risk factor for long-term mortality among RA patients [61].

Accumulating studies support the classic mucosal-origin hypothesis of RA. Gut mucosal microecological dyshomeostasis is regarded as an initiating factor that breaks immune tolerance and triggers autoimmune dysregulation in RA [62,63]. Clinical investigations have demonstrated significant structural alterations and reduced diversity of gut microbiota in RA-ILD patients, presenting a characteristic microecological dysbiosis phenotype [64]. Meanwhile, gut microbiota-driven bile acid metabolic disturbance modulates systemic inflammatory and immune homeostasis via multiple pathways and participates in the pathological process of RA-ILD, implying the potential regulatory role of the microbiota–metabolite axis in RA-ILD pathogenesis [65]. Pathogenically, gut dysbiosis can trigger local low-grade intestinal inflammation, disrupt mucosal barrier integrity, activate aberrant Th1/Th17 immune axes, and disturb intestinal mucosal immunity, thereby driving the abnormal production and release of RA-specific autoantibodies such as anti-CCP antibodies. Autoantibodies and activated immune cells disseminate widely via blood circulation and simultaneously target two critical mucosal immune sites: the joint synovium and lung tissue [66,67]. Notably, the lung also serves as a vital mucosal target for aberrant autoimmune activation in RA. Continuous infiltration of circulating autoimmune mediators into the pulmonary interstitium repeatedly induces chronic proliferative pulmonary inflammation and drives progressive fibrotic alterations. Accordingly, a characteristic pathological loop of the gut–lung axis is established: gut dysbiosis, mucosal immune imbalance, and RA-specific autoantibody production collectively trigger concurrent injury of joints and lungs, thereby accounting for the synchronized fluctuations between arthritis activity and interstitial pulmonary inflammation [61,68,69]. Intestinal barrier injury in RA-ILD patients mostly presents as chronic–persistent mild abnormalities without obvious organic intestinal lesions, dominated by functional hyper-permeability. Although existing evidence indicates that microbial dysbiosis such as Prevotella enrichment is closely associated with RA onset and articular inflammatory activity, and mediates systemic immune dysregulation and autoantibody generation in RA, direct and specific evidence linking gut microbiota or signature bacterial species to pulmonary lesions in RA-ILD remains insufficient. Most current microbiota-related studies focus on cohorts of patients with uncomplicated RA [70,71,72]. Specialized controlled studies specifically targeting RA-ILD populations are scarce, and the direct causal relationships between gut microecological disturbance and interstitial inflammation, fibrotic progression as well as pulmonary function decline have not been clarified. Therefore, the specific regulatory mechanisms of the gut–lung axis in RA-ILD and the clinical value of microbial biomarkers need further validation through large-sample, prospective specialized cohort studies.

### 3.3. SLE-ILD

SLE is a systemic autoimmune disease featured by chronic multi-organ inflammation and aberrant autoantibody production [73]. Among pulmonary manifestations in SLE patients, the most prevalent include pleuritis, pulmonary thromboembolism, and diffuse alveolar hemorrhage, together with the less common shrinking-lung syndrome. By contrast, SLE-ILD is relatively rare and mostly presents with an insidious onset and chronically progressive clinical phenotype [74]. Although substantial progress has been achieved in immunological research of SLE, its complete pathological regulatory network remains incompletely elucidated. Accumulating studies have identified a prominent “interferon signature” in SLE patients, characterized by sustained overexpression of type-I interferon-regulated genes, which serves as a core molecular hallmark driving systemic autoimmune dysregulation [75].

Multiple microbiome studies in recent years have demonstrated marked structural disturbance and characteristic abundance shifts of gut microbiota in SLE patients [76,77,78], providing preliminary theoretical evidence for the involvement of the gut–lung axis in pathological regulation of SLE-ILD. Nevertheless, no targeted studies have yet reported direct associations between microbiome dysbiosis and SLE-related pulmonary lesions, and investigations concerning the gut–lung axis in SLE-ILD remain at the theoretical–exploration stage. Based on existing theories and evidence from comparable CTD-ILD subtypes, SLE is presumed to possess a distinctive pattern of gut–lung axis disturbance, with moderate intermittent gut dysbiosis, fluctuating intestinal barrier impairment, and aberrant activation of type-I interferon pathways as potential subtype-specific hallmarks. By referencing research findings from other CTD subtypes, it is reasonable to hypothesize that, similar to the pathological mechanisms of SSc-ILD and RA-ILD, disrupted gut microbial community structure and microbial dyshomeostasis in SLE patients may perturb gut–lung axis homeostasis, indirectly modulate pulmonary autoimmune inflammatory activity, and contribute to the onset and chronic progression of SLE-ILD. To date, however, this pathological hypothesis of gut–lung axis-mediated SLE-ILD pathogenesis lacks direct experimental and clinical support. No studies have definitively established direct causal links between gut dysbiosis, intestinal barrier injury, and pulmonary inflammation as well as interstitial damage in SLE. Further high-quality studies are required to validate its precise molecular mechanisms, regulatory profiles, and clinical implications.

### 3.4. IIM-ILD

IIM-ILD represents a highly heterogeneous and life-threatening pulmonary complication of connective tissue diseases, mainly comprising dermatomyositis (DM), polymyositis (PM), and their autoantibody-defined subtypes [79]. Notably, anti-MDA5-positive DM patients are highly susceptible to rapidly progressive interstitial lung disease (RP-ILD), which can trigger extensive alveolar inflammation, acute lung injury, and even respiratory failure within a short period. It is currently regarded as the CTD-ILD subtype with the poorest prognosis and highest risk of acute exacerbation [80,81]. In dermatomyositis/polymyositis-associated interstitial lung disease (DM/PM-ILD), myopathy-induced injury of esophageal striated muscles leads to dysphagia and gastroesophageal reflux. On the one hand, microaspiration directly causes alveolar epithelial injury. On the other hand, persistent gastrointestinal inflammation disrupts gut microbial homeostasis and intestinal barrier integrity, induces metabolic disturbance and systemic autoimmune activation, and aggravates interstitial pulmonary inflammation and fibrotic progression via the gut–lung axis [82,83,84]. It is necessary to distinguish the physical injurious effect of gastroesophageal reflux from the systemic immune-metabolic regulatory mechanism mediated by the gut–lung axis. At present, clinical studies focusing on the gut–lung axis in DM/PM-ILD remain limited. Most existing studies merely report gut microecological abnormalities in IIM patients. Large-sample cohorts with which to validate quantitative correlations between gut microbiota, intestinal barrier biomarkers, and the progression rate of pulmonary lesions as well as risk of acute exacerbations are lacking. The causal relationship between gut dysbiosis and disease progression remains to be verified by prospective cohort studies.

### 3.5. pSS-ILD

pSS-ILD predominantly affects middle-aged women, and subclinical interstitial pulmonary lesions are frequently detected among this patient population [85]. Clinically, it is mostly characterized by chronic dry cough and exertional dyspnea, whereas rapidly progressive pulmonary fibrosis is relatively uncommon [86]. In terms of imaging and pathological phenotypes, non-specific interstitial pneumonia (NSIP) represents the most prevalent subtype of pSS-ILD, while lymphocytic interstitial pneumonia (LIP) is regarded as a relatively distinctive pulmonary injury phenotype for pSS [87]. Typical HRCT manifestations include diffuse ground-glass opacities and multiple thin-walled pulmonary cysts. Moreover, such patients carry a potential risk of long-term lymphoma transformation, which requires long-term clinical follow-up and surveillance [88].

Gastrointestinal involvement in pSS is pathologically featured by mucosal gland atrophy and chronic lymphocytic infiltration of the digestive tract, frequently accompanied by dysphagia, dyspepsia, and gastroesophageal reflux. Its gastrointestinal injury mechanism exhibits prominent subtype-specific differences compared with smooth-muscle fibrosis in systemic sclerosis and esophageal striated-muscle injury in inflammatory myopathy [89,90]. Compared with dcSSc-ILD, direct pulmonary injury induced by microaspiration secondary to gastroesophageal reflux plays a relatively limited role in pSS-ILD. Studies have identified Th17 cells and their signature cytokine IL-17 within salivary gland lesions of pSS patients [91]. Chronic gastrointestinal inflammation driven by pSS can trigger intestinal barrier impairment and gut dysbiosis. Such intestinal abnormalities may contribute to interstitial inflammatory activation and disease progression in pSS-ILD. Nevertheless, current evidence remains correlative, and specific causal regulatory mechanisms need further validation. Dedicated investigations addressing the modulatory role of the gut microbiome in the onset and progression of pSS-ILD are still scarce, and systematic relevant studies are lacking.

**Table 1:** Evidence for gut–lung axis involvement across CTD-ILD subtypes.

CTD-ILD Subtype	Symptoms of Gut Microbiome Dysbiosis	Damage to the Intestinal Barrier	Circulating Microenvironment Medium	Pulmonary Immunity and Fibrosis	References
Systemic sclerosis-associated interstitial lung disease (SSc-ILD)	Increased Bacteroidetes/Teribactria ratio; elevated abundances of *Arcobacter*, *Ruminococcus*, and *Prevotella*; and depleted butyrate-producing taxa including *Faecalibacterium* and *Clostridium*. Compared with SSc patients without ILD, feces from SSc-ILD patients contain higher levels of *Anaerotignum faecicola* and *Roussiella hominis*.	Elevated fecal calprotectin is independently associated with ILD. Small intestinal bacterial overgrowth (SIBO) and dysmotility are common; intestinal permeability findings inconsistent. Some studies have reported that serum zonulin levels correlate with FVC and DLCO.	Reduced fecal/serum butyrate and butyrate-producing bacteria. Reduced SCFAs (especially butyrate). Metabolic shifts in amino acid and bile acid pathways.	No human lung microbiome studies have specifically examined SSc-ILD.	[14,92,93,94]
Rheumatoid arthritis-associated interstitial lung disease (RA-ILD)	Increased *Escherichia/Shigella* and *Streptococcus*. Decreased *Roseburia*, *Ruminococcus*, *Slackia,* and *Paraprevotella*. A trend toward reduced *Faecalibacterium*.	Elevated serum LPS and LBP levels in RA-ILD, suggesting increased intestinal permeability. LPS is associated with ILD risk.	Elevated serum LPS and LBP levels. Reduced SCFA producers. Reduced levels of butyrate and other anti-inflammatory metabolites.	BALF microbiota showed reduced diversity and enrichment of oral-originated taxa. BALF microbial changes correlate with RA severity.	[95,96]
Systemic lupus erythematosus-associated interstitial lung disease (SLE-ILD)	Reduced gut microbiota diversity in SLE. Increased Proteobacteria, *Rhodococcus*, *Enterococcus gallinarum*, *Eggerthella*, *Klebsiella*, *Prevotella*, *Ruminococcaceae*, and *Flavonifractor*. Decreased *Firmicutes*, *Dialister*, and *Pseudobutyrivibrio*. Active SLE: Enrichment of *Escherichia-Shigella* and other pathobionts.	Leaky gut: elevated serum zonulin and fecal calprotectin. Elevated serum sCD14 and LPS levels, indicating bacterial translocation. Active SLE: zonulin linked to Tfh/Tfr imbalance. *Ruminococcus gnavus* enhances intestinal permeability.	Elevated circulating LPS, sCD14, and zonulin levels. Reduced SCFA-producing bacteria and anti-inflammatory metabolites. Bacterial translocation to mesenteric lymph nodes and visceral organs. *E. gallinarum* can induce anti-β2GP1 antibody production.	No human lung microbiome studies have been conducted specifically in SLE-ILD.	[97]
Idiopathic inflammatory myopathy-associated interstitial lung disease (IIM-ILD)	Reduced fecal microbiota diversity in DM. ILD-related myositis-specific autoantibody (MSA) subgroups showed lower diversity and distinct composition. Increased abundance of Bacteroides/Firmicutes and *Proteobacteria* and decreased abundance of Christensenellaceae and Ruminococcaceae. The ILD-MSA subgroup was enriched in *Proteobacteria* and LPS synthesis/transport pathways.	Direct human data are limited. Enhanced LPS-related metabolic pathways in the ILD-MSA subgroup suggest possible barrier dysfunction.	Enhanced LPS synthesis/transport pathways and possible elevated circulating LPS (indirect evidence). Reduced SCFA-producing bacteria.	Patients with IIM-ILD had elevated levels of *Pseudomonas* spp., *Bacillus* spp., and *Pseudomonas aeruginosa* in their bronchoalveolar lavage fluid (BALF); Bacillus spp. were positively correlated with KL-6; *Pseudomonas aeruginosa* was associated with IL-2, IL-8, and IgM; and there were differences in the BALF microbiome between patients with DM-ILD and RA-ILD.	[98,99]
Primary Sjögren’s syndrome-associated interstitial lung disease (pSS-ILD)	Reduced gut microbiota richness and evenness.	Increased intestinal permeability was confirmed using the lactulose/mannitol absorption test. Elevated serum zonulin levels and *Prevotella copri* abundance positively correlated with zonulin levels. Elevated fecal calprotectin, reflecting intestinal wall inflammation Reduced FOXP3 expression, Treg dysfunction, and Th17/Treg imbalance.	Phenylacetic acid (PAA): L. salivarius metabolizes L-phenylalanine to PAA, which is systemically absorbed and reaches the lungs. Reduced SCFA-producing bacteria, decreased butyrate. Elevated systemic pro-inflammatory cytokines: IL-6, IL-12, IL-17, and TNF-α. Elevated circulating zonulin.	No dedicated lung microbiome studies have been conducted in pSS-ILD.	[100,101,102]

## 4. Clinical Application Value of Microbiota–Gut–Lung Axis in CTD-ILD

CTD-ILD is characterized by insidious onset, fluctuating disease activity, high risk of acute exacerbation, and substantial individual heterogeneity in prognosis. Current clinical diagnosis, disease evaluation, and follow-up monitoring mainly rely on chest high-resolution CT, pulmonary function tests, and traditional serum fibrotic biomarkers such as KL-6 and SP-D [103,104]. However, these conventional modalities have inherent limitations, including radiation exposure, high medical costs, insufficient sensitivity for early lesions, limited specificity, and delayed evaluation efficacy, which restrict their performance in the early identification and precise dynamic monitoring of CTD-ILD. Emerging studies have demonstrated that dysregulation of the microbiota–gut–lung axis participates in inflammatory activation and fibrotic progression of CTD-ILD, and gut microecological disturbance, metabolic abnormalities, and intestinal barrier injury can mediate pulmonary damage through systemic immune-inflammatory responses [14,92]. Nevertheless, relevant investigations remain in the preliminary exploratory stage, and a mature clinical application system has not yet been established. This section systematically summarizes the research progress of the gut–lung axis in CTD-ILD from four dimensions: potential evaluation value, disease early warning exploration, targeted intervention strategies, and traditional Chinese medicine (TCM) characteristic intervention, and further analyzes the current limitations of its clinical application.

### 4.1. Potential Diagnostic and Disease Evaluation Value of Gut–Lung Axis-Related Biomarkers

Gut–lung axis dysregulation drives the onset and progression of CTD-ILD. Intestinal microecological abnormalities, metabolic disturbances, and intestinal barrier dysfunction can indirectly reflect the status of pulmonary inflammation and fibrosis, endowing gut–lung axis components with promising potential as novel auxiliary biomarkers for disease evaluation. Compared with thoracic imaging examinations and conventional serological markers, gut–lung axis-related biomarkers exhibit distinct advantages including convenient sampling, non-invasiveness, and feasible dynamic repeated detection. These biomarkers can reveal pathological alterations at the upstream immune-metabolic level, thereby compensating for the deficiencies of traditional evaluation systems, which primarily reflect end-stage pulmonary damage and lack sufficient sensitivity for early lesions.

Current relevant studies primarily focus on four major research directions, namely gut microbial characteristics, metabolic signatures, intestinal barrier biomarkers, and multi-omics predictive models, while their diagnostic efficiency and clinical applicability remain under exploratory investigation. Distinct CTD-ILD subtypes exhibit specific gut dysbiosis phenotypes, which hold great potential for subtype differentiation and disease stratification. The overall severity of gut microbial disturbance is correlated with pulmonary lesion degree and inflammatory activity, providing valuable references for the pathological evaluation of CTD-ILD. Microbial metabolites serve as crucial mediators bridging gut microecological imbalance and remote pulmonary injury. Multiple pivotal metabolites, including short-chain fatty acids, bile acids, tryptophan metabolites, and lipopolysaccharides, can sensitively reflect homeostatic disruption of the gut–lung axis [105]. Decreased levels of beneficial metabolites such as short-chain fatty acids are closely associated with impaired intestinal immune regulation and aggravate systemic low-grade inflammation, whereas elevated endotoxin levels represented by lipopolysaccharides indicate exacerbated microecological disorder and increased risk of bacterial translocation. These metabolic abnormalities correspond to subclinical pulmonary inflammation and early fibrotic alterations, theoretically enabling the early identification of occult pulmonary lesions. Intestinal barrier damage acts as a core initiating factor for pathological gut–lung axis activation. Abnormal levels of intestinal barrier biomarkers are correlated with the scope of pulmonary involvement, fibrosis severity, and the declining trend of pulmonary function, which can serve as auxiliary indicators to reflect the overall damage degree of the gut–lung axis [106]. To address the insufficient stability and limited accuracy of single biomarkers, multi-omics predictive models have been tentatively constructed by integrating gut microbial profiles, metabolomic features, intestinal barrier indices, and clinical parameters.

Despite the promising research potential of the above-mentioned gut–lung axis biomarkers, their clinical value for disease evaluation remains constrained by prominent limitations, and none of them have been applied in routine clinical practice. Gut microbial and metabolic profiles are highly susceptible to multiple confounding factors, including diet, medication, antibiotic exposure, regional and ethnic differences, and underlying gastrointestinal disorders. Most existing studies fail to adequately control these confounders, resulting in high heterogeneity of research findings [63,92]. Single biomarkers lack sufficient disease specificity, as intestinal microecological and barrier disturbances are not unique pathological alterations in CTD-ILD, making it difficult to independently distinguish disease subtypes or evaluate inflammatory activity. Furthermore, current multi-omics models are still in the preliminary exploratory stage, characterized by small sample sizes, insufficient external validation, and inadequate confounding adjustment, and their stability and generalization performance require further improvement. Overall, existing gut–lung axis biomarkers are currently limited to basic research. Standardized, large-sample cohort studies are urgently needed to clarify their authentic efficacy for clinical evaluation in the future.

Beyond protein and metabolite biomarkers, circulating circular RNAs (circRNAs) have emerged as stable liquid biopsy candidates; generated by back-splicing and resistant to degradation, they regulate pulmonary fibrosis via microRNA sponging, TGF-β/Smad signaling, and fibroblast-to-myofibroblast differentiation [107]. Complementary integrative multi-omics can reconstruct the gut–lung axis regulatory network and improve subtype stratification [108]. In parallel, label-free Raman spectroscopy and surface-enhanced Raman scattering (SERS) can non-destructively acquire the molecular fingerprint of serum, bronchoalveolar lavage fluid, or lung tissue, and, combined with machine learning, distinguish fibrotic grading with high accuracy, offering a rapid, repeatable complement to molecular biomarkers for monitoring CTD-ILD-related pulmonary fibrosis [109].

### 4.2. Early Warning Value of Gut–Lung Axis Dysregulation for Disease Progression and Acute Exacerbation

Gut microecological disturbance and intestinal barrier injury effectively reflect aberrant activation of the microbiota–gut–lung axis, exhibiting potential early warning value for disease progression, pulmonary function deterioration, and long-term adverse prognosis in CTD-ILD. Compared with traditional inflammatory indicators and static imaging evaluation, gut–lung axis-related biomarkers can capture subclinical cross-organ inflammatory signals and early fibrotic initiation at an earlier stage, conferring unique application potential for early warning, dynamic monitoring, and individualized risk stratification of CTD-ILD. Existing exploratory studies have demonstrated that disease severity is closely correlated with the degree of gut microecological imbalance in CTD-ILD patients; specifically, more severe microecological dyshomeostasis corresponds to a more progressive deterioration of pulmonary lesions [14,110]. Several studies have reported that progressive gut dysbiosis is significantly associated with the continuous decline of pulmonary function parameters, including forced vital capacity (FVC) and diffusing capacity of the lung for carbon monoxide (DLCO) [111]. Nevertheless, restricted by the limited scale and quality of current evidence, microbial signatures cannot yet reliably distinguish stable from rapidly progressive CTD-ILD phenotypes, and only provide preliminary clues for interpreting the heterogeneous disease trajectories of CTD-ILD.

Intestinal barrier dysfunction indicators can further quantify the pro-inflammatory and pro-fibrotic activities mediated by the gut–lung axis, with promising predictive efficacy for the acute exacerbation of CTD-ILD [112]. Gut dysbiosis increases intestinal mucosal permeability, leading to significant upregulation of circulating intestinal barrier injury biomarkers such as zonulin and lipopolysaccharide [113]. The translocation of gut-derived endotoxins into the systemic circulation disrupts pulmonary immune and inflammatory homeostasis, induces the release of pro-inflammatory and pro-fibrotic mediators including IL-6, IL-17, and TGF-β, and consequently aggravates alveolar epithelial injury and interstitial remodeling [114,115]. Based on the pathological mechanisms of the gut–lung axis, it is speculated that peripheral intestinal barrier biomarkers may increase abnormally prior to the onset of CTD-ILD acute exacerbation. Therefore, multi-dimensional evaluation integrating gut microbial characteristics and intestinal barrier indicators is expected to improve the predictive ability for acute exacerbation risk, compensating for the limited capacity of conventional clinical indicators in identifying subclinical disease fluctuations. This strategy provides a novel direction for early intervention, individualized management, and long-term disease control of CTD-ILD. Although gut–lung axis biomarkers display favorable predictive potential, their clinical warning value remains restricted by multiple limitations at the current stage and cannot meet the criteria for routine clinical application.

### 4.3. Targeted Intervention Strategies Based on the Gut–Lung Axis

In recent years, the pathological regulatory mechanisms underlying the gut–lung axis have opened new perspectives for adjuvant therapy of CTD-ILD. Gut microecological disturbance not only disrupts intestinal mucosal barrier integrity to facilitate translocation of bacterial endotoxins and metabolites into the bloodstream, thereby activating pulmonary innate and adaptive immune responses, but also indirectly contributes to the initiation and progression of pulmonary fibrosis by modulating signaling molecules such as short-chain fatty acids and tryptophan metabolites [5,116,117]. This mechanistic insight challenges the traditional view that CTD-ILD represents merely a local pulmonary disorder, and identifies the gut as an upstream driver of systemic inflammatory and fibrotic cascades. Accordingly, targeted strategies to remodel gut microbiota, restore intestinal barrier function, and block gut-derived pro-inflammatory/pro-fibrotic pathways are regarded as promising approaches to overcome the limitations of current anti-inflammatory and ant-fibrotic agents, including suboptimal therapeutic efficacy and substantial adverse-effect profiles.

In terms of translational prospects, advances in metagenomics, metabolomics, and single-cell sequencing enable high-resolution characterization of compositional and functional dysbiosis of gut microbiota in patients with CTD-ILD, providing technical support for biomarker discovery and identification of potential therapeutic targets. Current intervention strategies derived from gut–lung axis research mainly include dietary modulation, microbiota-based interventions, metabolite-targeted therapy, fecal microbiota transplantation (FMT), and immune pathway modulation [5,118,119,120,121,122]. These approaches have demonstrated certain anti-inflammatory and anti-fibrotic potential in pre-clinical models of multiple pulmonary diseases as well as small-sample exploratory clinical studies. In particular, FMT and supplementation with specific metabolites may achieve long-term restoration of microbial homeostasis by reconstructing the structure and function of gut microbial communities, showing superior therapeutic potential compared with conventional microbiota-modulating regimens. Based on the levels of available evidence, this section hierarchically summarizes the current status, mechanistic features, and existing drawbacks of each intervention strategy. The mechanisms, research evidence, and translational barriers of these therapeutic modalities are summarized in Table 2.

At present, multiple bottlenecks hinder the clinical translation of gut–lung axis-targeted intervention strategies [5,120,140]. First, most relevant studies are small-sample exploratory investigations, lacking high-level evidence from large-scale randomized controlled trials. In addition, considerable heterogeneity exists across studies regarding detection modalities and intervention protocols, leading to limited reproducibility of research outcomes. Second, the precise molecular mechanisms underlying cross-organ signal regulation of the gut–lung axis in patients with CTD-ILD remain incompletely elucidated, which impedes the development of precise targeted interventions. Meanwhile, unified clinical implementation standards are absent for regimens including probiotic administration, fecal microbiota transplantation, and metabolite-targeted therapy. Data concerning dosage, treatment duration, eligible populations, and long-term safety are still scarce, and therapeutic responses vary substantially among individuals. Taken together, gut–lung axis-based interventions are currently confined to basic exploratory and auxiliary research stages and cannot replace standard-of-care therapies such as glucocorticoids, immunosuppressants, and anti-fibrotic agents. Future efforts should rely on standardized prospective cohort studies and harmonized intervention protocols, combined with integrated analysis of mechanistic insights and clinical phenotypes, to further define the clinical utility and suitable target populations for gut–lung axis-directed interventions in CTD-ILD.

### 4.4. TCM-Specific Therapeutic Strategies Based on “Simultaneous Treatment of Lung and Intestine”

TCM interventions guided by the principle of “simultaneous treatment of lung and intestine” can ameliorate pathological injuries in CTD-ILD by restoring gut–lung axis homeostasis and targeting multi-dimensional pathological pathways, representing a distinctive research direction for integrated Chinese–Western medicine in the prevention and management of CTD-ILD. Modern pharmacological and mechanistic studies have validated that multiple TCM compound prescriptions and monomeric components exert dual lung–intestine regulatory effects by reshaping gut microbial composition, repairing intestinal barrier function, correcting microbial metabolic dysregulation, and suppressing aberrant inflammatory and fibrotic signaling cascades [141,142,143,144]. From the perspective of microecological and metabolic modulation, TCM interventions can optimize gut microbiota profiles, enrich the abundance of beneficial commensals, boost the production of protective metabolites such as short-chain fatty acids, and reduce the levels of gut-derived harmful metabolites including lipopolysaccharides, thereby mitigating gut-driven systemic inflammatory activation [145,146]. For intestinal barrier restoration, bioactive TCM ingredients can upregulate the expression of intestinal tight-junction proteins, reduce intestinal permeability, and alleviate gut-leak-associated injury, which blocks the translocation and infiltration of gut-originated inflammatory mediators into the lung at the source [147,148]. At the downstream signaling level, TCM agents can attenuate pulmonary inflammatory infiltration and interstitial remodeling, slow fibrotic progression, and improve clinical symptoms as well as pulmonary function by suppressing excessive NF-κB-driven inflammatory responses, inhibiting hyper-activated TGF-β/Smad fibrotic signaling, and rebalancing the Th17/Treg immune axis [149]. Compared with single-target-oriented Western drug interventions, the multi-component and multi-pathway regulatory property of TCM is better adapted to the multi-layered dyshomeostasis of the gut–lung axis, conferring unique therapeutic potential for disease management during the chronic phase of CTD-ILD.

## 5. Discussion

Accumulating evidence indicates that immune dysregulation, persistent activation of chronic inflammation, and progressive interstitial pulmonary fibrosis constitute the core pathological features underlying the onset and development of CTD-ILD. As a critical cross-organ regulatory pathway, gut–lung axis dyshomeostasis is hypothesized to mediate the full-spectrum pathological evolution of CTD-ILD. Through cross-organ crosstalk within the microbiota–gut–lung axis, gut dysbiosis, intestinal barrier disruption, gut-derived inflammation, and metabolic abnormalities can trigger the imbalance of the pulmonary immune microenvironment via the circulatory system, and further drive inflammatory infiltration and fibrotic remodeling in lung tissue. This core pathogenic mechanism has been partially validated [117,150]. In terms of subtype-specific evidence among connective tissue diseases, sufficient data support the association between gut–lung axis disturbance and SSc-ILD. Multiple gut-microbiota-targeted studies have confirmed that its dysregulated microbial, metabolic, and immune signatures are highly concordant with interstitial lung injury. By contrast, evidence for gut–lung axis-mediated regulation in RA-ILD, SLE-ILD, IIM-ILD, and pSS-ILD remains largely indirect. Direct molecular mechanistic evidence and clinical correlative data for these subtypes are still urgently needed.

Although the pathogenic role of the gut–lung axis in CTD-ILD has been gradually recognized, many scientific questions in this field remain unresolved, and no unified consensus has been reached. The primary controversy lies in ambiguous causal relationships. Most existing studies have merely demonstrated prominent gut dysbiosis and intestinal barrier impairment in CTD-ILD patients, yet cannot distinguish whether gut microecological disturbance acts as an initiating trigger for CTD-ILD, a secondary pathological alteration secondary to disease progression, or a pivotal intermediate amplifier that augments pulmonary inflammation and fibrosis. Direct experimental evidence supporting upstream–downstream causal regulatory relationships is still lacking. Furthermore, the shared and subtype-specific mechanisms of gut–lung axis regulation across different CTD subtypes remain debatable. It remains unclear whether universal core pathogenic bacteria, metabolic pathways, and immune regulatory networks exist across subtypes, and subtype-specific dysregulated targets are yet to be identified.

Current basic research findings concerning the gut–lung axis cannot be efficiently translated into clinical practice for CTD-ILD diagnosis and management, and multiple objective bottlenecks hamper translational progress. Large-sample longitudinal cohorts with long-term follow-up are scarce. A complete clinical evidence chain describing dynamic gut–lung axis alterations across the disease continuum—including CTD without ILD, early-stage occult ILD, progressive ILD, and severe fibrotic ILD—is still absent. Accordingly, quantitative correlations between gut–lung axis dysregulation and disease stage, disease activity, and risk of acute exacerbation cannot be firmly established. Secondly, gut–lung axis-related biomarkers (microbial profiles, metabolites, and intestinal barrier indicators) lack unified detection protocols, quality control criteria, and clinical cut-off values. Substantial discrepancies exist among detection systems applied in different studies, resulting in poor reproducibility and hindering cross-study comparison. Meanwhile, most multi-omics-derived candidate biomarkers remain at the preliminary exploratory stage, without external cohort validation or clinical performance verification; their diagnostic and prognostic values have not been widely acknowledged. In addition, targeted interventional studies focusing on the gut–lung axis are limited. Key clinical parameters, including intervention timing, dosage, and treatment regimens, have not been standardized. The clinical suitability of integrated Chinese–Western therapeutic strategies also lacks systematic validation, which impedes standardized clinical implementation.

Given the current research landscape and existing limitations, future investigations should prioritize mechanistic exploration and clinical translation. Systematic and standardized high-quality studies are required to translate gut–lung axis theories toward individualized clinical management of CTD-ILD. For clinical cohort construction, multicenter, large-sample CTD-ILD-specific longitudinal cohorts should be established, enrolling patients across diverse subtypes and disease stages. Biological specimens and clinical data, including stool samples, serum, bronchoalveolar lavage fluid, sputum, chest HRCT, and pulmonary function parameters, should be collected under standardized procedures to build comprehensive disease-oriented databases. For mechanistic research, multi-omics approaches including 16S rRNA gene sequencing, metagenomic sequencing, untargeted/targeted metabolomics, transcriptomics, and immune profiling should be integrated to systematically dissect subtype-specific molecular regulatory networks of gut–lung axis disturbance. Core biomarkers and pathogenic targets for each subtype should be screened, and the functional mechanisms of bioactive components from TCM regimens for simultaneous lung–intestine treatment should be elucidated. For clinical translation, multicenter prospective studies are needed to unify detection standards and diagnostic thresholds for candidate biomarkers, and to construct non-invasive models for early warning, disease assessment, and prognosis prediction based on the gut–lung axis. Meanwhile, high-quality randomized controlled clinical trials should be performed to systematically validate the adjuvant therapeutic efficacy of probiotics, postbiotics, and TCM compound prescriptions for simultaneous lung–intestine therapy. Individualized intervention strategies should be optimized, and the applicable scenarios, optimal intervention timing, long-term efficacy, and safety of different interventions should be clarified. Collectively, these efforts will provide novel insights and theoretical foundations to overcome therapeutic obstacles and improve long-term clinical outcomes for patients with CTD-ILD.

## 6. Conclusions

Advancing research on the microbiota–gut–lung axis has provided innovative perspectives and theoretical foundations for systematically elucidating the complex immune-metabolic disorders and subtype heterogeneity underlying CTD-ILD. Although accumulating evidence supports its mechanistic and translational potential, several critical shortcomings remain in this field. Current studies have only confirmed correlative relationships between gut–lung axis dysregulation and CTD-ILD, whereas direct causal evidence is still insufficient. Furthermore, this field lacks large-sample longitudinal cohort data, unified biomarker evaluation criteria, and high-quality clinical intervention trials. In addition, the material basis and core therapeutic targets of TCM interventions targeting the gut–lung axis remain to be systematically clarified.

Future studies should focus on standardization and precision. Integrated multi-omics techniques, standardized cohort construction, and precise intervention verification are required to clarify the authentic value of the gut–lung axis for early warning, subtyping, and adjuvant treatment of CTD-ILD. Systematic validation of the gut–lung axis in early risk prediction, accurate subtype classification, and individualized adjuvant therapy will help establish an updated diagnostic and therapeutic system for CTD-ILD, providing reliable strategies to improve long-term-patient prognosis.

## Figures and Tables

**Table 2:** Intervention strategies targeting the gut–lung axis in CTD-ILD: mechanisms, evidence, and clinical translation barriers.

Intervention Strategy	Mechanistic Evidence	Research Evidence in CTD/CTD-ILD	Clinical Translation Barriers	References
**Probiotics** e.g., *Lactobacillus*, *Bifidobacterium*, *Clostridium butyricum*, etc.	*Bifidobacterium* promotes the generation of Th1 and Treg cells by modulating T-cell polarization, thereby cooperatively inducing immune tolerance. Strains such as Lactobacillus lactis and *Bacillus longus* reduce TNF-α and IL-6 levels, preserve epithelial barrier integrity, and improve pulmonary function parameters. *Clostridium butyricum* facilitates SCFA production and exerts beneficial effects on intestinal metabolic processes. It modulates the gut microbiota, boosts the biosynthesis of SCFAs and antioxidant metabolites, and synthesizes specific tryptophan metabolites to enhance antioxidant and anti-inflammatory activities. These effects suppress M1 macrophage polarization and alleviate pulmonary oxidative stress and inflammation.	*Bifidobacterium longum* RAPO alleviates skin sclerosis and pulmonary fibrosis in bleomycin-induced SSc mice via macrophage reprogramming and SCFA elevation. Supplementation with multi-strain probiotics reduces fecal calprotectin and improves intestinal dysmotility in patients with SSc/pSS; partially decreases serum KL-6 and systemic inflammatory markers. Meta-analyses of autoimmune cohorts show modest reductions in disease activity in SLE, pSS, and SSc, but inconsistent effects in RA.	Probiotic effects exhibit strong strain specificity, and no standardized probiotic formulations have been validated specifically for distinct CTD-ILD subtypes. No human randomized controlled trials (RCTs) dedicated to CTD-ILD are available; all human-derived data originate from studies involving CTD patients without pulmonary-related endpoints. Clinical trials on probiotics lack standardized, lung-specific outcome measures.	[14,105,123,124,125,126,127]
**Prebiotics and dietary fiber** e.g., *dietary fiber, fructo-oligosaccharides (FOS), galacto-oligosaccharides (GOS), and TCM polysaccharides (Astragalus, Lycium barbarum)*	Selectively fuel butyrate-producing bacteria to boost endogenous intestinal SCFA synthesis, strengthen intestinal mucosal barrier, and reduce bacterial translocation. Circulating butyrate suppresses NF-κB and TGF-β pathways in lung tissue, thereby restraining fibroblast proliferation and extracellular matrix deposition. Gut microbial dysbiosis, augmented intestinal permeability, and Tfh/Tfr homeostatic imbalance in active SLE.	A high-fiber diet generates butyrate, which confers anti-fibrotic effects against bleomycin-induced pulmonary fibrosis. Astragalus and Lycium polysaccharides enrich Lactobacillus and Bifidobacterium while suppressing the TLR4/NF-κB signaling pathway. High-fiber dietary intervention lowers intestinal inflammation biomarkers (fecal calprotectin, serum zonulin) in patients with RA and pSS and reduces SIBO prevalence in SSc cohorts.	No CTD-ILD human RCTs. Difficult to standardize dietary interventions and poor long-term compliance. Large interindividual baseline differences in the gut microbiota result in high heterogeneity in prebiotic fermentation efficiency.	[97,128,129,130,131,132]
**Postbiotics** SCFAs (butyrate, acetate, and propionate), bacterial lysates, extracellular vesicles (EVs), and pasteurized *Akkermansia muciniphila*	Active components (butyrate, bacterial polysaccharides, and peptidoglycans) directly inhibit zonulin release to reverse intestinal permeability defects without relying on live strain colonization. Blocking the LPS-TLR4 systemic inflammatory cascade reduces circulating IL-17 and TNF-α independent of gut flora remodeling. SCFAs modulate bone marrow hematopoiesis to shape the pool of lung-targeted dendritic cell and macrophage precursors.	Butyrate postbiotics and heat-inactivated *Bifidobacterium* extracts significantly attenuated lung and skin fibrosis in SSc murine models, independent of live microbiota viability. Butyrate improves intestinal barrier function and reduces bacterial translocation.	A lack of standardized manufacturing and quality control protocols exist for bioactive postbiotic components. Synergistic or antagonistic interactions between postbiotics and routine immunosuppressive therapies for connective tissue diseases have not been well defined. Preclinical data alone support its anti-fibrotic activity, whereas robust clinical evidence is lacking.	[62,94,128,131,133,134,135]
**Fecal microbiota transplantation (FMT)**	Globally reshapes the community structure of gut microbiota, elevates the Firmicutes/Bacteroidetes ratio, and restores the depleted α-diversity of gut microbiota in patients with CTD-ILD. Fecal microbiota transplantation inhibits NF-κB phosphorylation and reduces the release of pulmonary pro-inflammatory cytokines. FMT can improve airway function, restore lung tissue pathology, alleviate alveolar wall thickening, reduce inflammatory cell infiltration, and markedly decrease collagen fiber deposition.	FMT from healthy donor mice restores gut SCFA pools, reduces lung interstitial inflammation, and collagen deposition in bleomycin-induced pulmonary fibrosis and lupus-prone murine models. Rare refractory RA and SLE case series report transient improvements in intestinal inflammation and disease activity scores post-FMT.	Standardized protocols for donor screening, microbiota matching, and pre-transplant bowel preparation dedicated to connective tissue diseases are lacking, leading to substantial inter-donor variability in therapeutic outcomes. Safety concerns persist: immunocompromised hosts are at risk of transmission of opportunistic pathogens and strains with unknown virulence.	[32,116,136,137,138,139]

## Data Availability

No new data were created or analyzed in this study. Data sharing is not applicable to this article.
